# Case Report: a novel missense variant of *FGD5* in a family with tetralogy of Fallot

**DOI:** 10.3389/fgene.2025.1663959

**Published:** 2025-10-23

**Authors:** Meng-Wei Liu, Yi Dong, Rong Xiang, Liping Wu

**Affiliations:** ^1^ Longgang District Maternity and Child Healthcare Hospital of Shenzhen City (Affiliated Shenzhen Women and Children’s Hospital (Longgang) of Shantou University Medical College), Shenzhen, China; ^2^ College of Basic Medical, Xinjiang Medical University, Urumqi, China; ^3^ School of Life Sciences, Central South University, Changsha, China

**Keywords:** tetralogy of Fallot, FGD5, genetic variant, whole-exome sequencing, irregular dominance

## Abstract

**Background:**

Congenital heart disease (CHD) is among the most prevalent birth defects in newborns, with tetralogy of Fallot (TOF) being a classic example. Within weeks to months after birth, infants with TOF often exhibit cyanosis of the skin, lips, or nails and respiratory distress. Early medical intervention is crucial to enhance outcomes and ensure a better long-term prognosis.

**Methods and results:**

A young Chinese couple was referred for prenatal counseling at a gestational age of 26^+3^ weeks for fetal complex CHD, including pulmonary artery stenosis, a widened aorta with overriding, and absence of the ductus arteriosus in their affected fetus, who was later diagnosed with TOF. To determine the genetic basis of the congenital heart defect, whole-exome sequencing and Sanger sequencing were performed to identify potential pathogenic variants. Subsequently, a heterozygous missense variant (c. 3233C>A, p.T1078K) of *FGD5* was identified in both the affected fetus and its unaffected carrier mother, demonstrating an inheritance pattern with irregular dominance. This variant is exceptionally rare in public genomic databases and determined to be the genetic cause of TOF in the proband.

**Conclusion:**

We identified a novel heterozygous missense variant of *FGD5* in a Chinese family with TOF, which expands the genetic spectrum of the disease.

## Introduction

Congenital heart disease (CHD), defined as structural malformations of the heart and major intrathoracic vessels, affects approximately 1% of live births worldwide, making it the most common category of congenital anomalies (Bouma and Mulder, 2017). As a major cyanotic CHD subtype, tetralogy of Fallot (TOF) demonstrates heritable components (Sierant et al., 2025). TOF (OMIM #187500), a complex congenital heart malformation primarily influenced by genetic factors, was first described by French pathologist Étienne-Louis Arthur Fallot in the late 19th century. Fallot defined four core anatomical features: right ventricular outflow tract obstruction (pulmonary stenosis), ventricular septal defect, overriding of the aorta, and hypertrophy of the right ventricle, which emerge as a whole complex deformity that is the main cause of severe cyanosis of the skin and mucous membranes (O'Brien and Marshall, 2014). Several gene variants known to be associated with the development of this genetic disorder include *NKX2*.*5*, *GATA4*, *GATA6*, *ZFPM2*, and *JAG1* (Goldmuntz et al., 2001; Pizzuti et al., 2003; Zhang et al., 2009; Kola et al., 2011; Huang et al., 2013).

*FGD5* (FYVE, RhoGEF, and PH domain containing 5, MIM*614788), a member of the FGD family and located on chromosome 3p25.1, contains 21 exons encoding a protein of 1,462 amino acids. This endothelium-specific gene functions as a guanine nucleotide exchange factor (GEF) in vascular endothelial cells to regulate CDC42 activity and participate in angiogenesis (Cheng et al., 2012; Kurogane et al., 2012). FGD5 was first associated with TOF in 2019 (Reuter et al., 2019).

In this study, we present the case of a male fetus prenatally diagnosed with TOF exhibiting hallmark features of pulmonary stenosis, ventricular septal defect, and aortic overriding. An *FGD5* missense variant (NM_152536.4: c. 3233C>A, p.T1078K; chr3:14860469C>A/hg19) was identified in the male fetus, and its pathogenicity was analyzed using three-dimensional protein modeling. This study expands the *FGD5* variant spectrum, demonstrates the pathogenicity of Dbl homology (DH)-domain variants in TOF, and elucidates their role in disrupting cardiovascular development. As TOF has previously been considered a genetic disease with high penetrance (Wang et al., 2022), the discovery of asymptomatic carriers in the family harboring the same p.T1078K variant made the diagnosis challenging.

## Materials and methods

### Subjects

A fetus (index case II-1) exhibiting ultrasound abnormalities at 22 weeks’ gestation and two asymptomatic parents from a Han Chinese family were enrolled. Peripheral blood samples were collected from parents (I-1 and I-2), while the aborted fetus from the proband was obtained for genetic analysis. Whole-exome sequencing (WES) revealed the same missense variant in the proband and the mother. This study received ethical approval from the School of Life Sciences of Central South University in compliance with the Declaration of Helsinki, and written parental consent was obtained.

### DNA extraction, whole-exome sequencing, and analysis

Genomic DNA was extracted from whole peripheral blood (EDTA-K_2_ anticoagulated) using the QIAamp DNA Blood Mini Kit (QIAGEN, United States). Variants were annotated using MutationTaster, PolyPhen-2, SIFT, CADD, REVEL, and population databases (gnomAD and 1000 Genomes) (Fan et al., 2019).

### Variant validation

The *FGD5* variant (NM_001320276.2: exon9: c. 3233C>A: p.T1078K) and its flanking sequences were obtained from NCBI (https://www.ncbi.nlm.nih.gov/gene/152273). The PCR primers were designed by the Ensemble genome browser and PrimerQuest to amplify the exon sequences provided by Sangon Biotech (Shanghai, China). PCR products were approximately 500 bp in length. The primer sequences were as follows: F: 5′-CAA​GTG​CTC​CTC​ACA​GAC​TAT​TT-3′ and R: 5′-CAT​AGC​GTG​GAG​ATA​CGA​GAG​A-3′; for c. 3233C>A, we amplified a 541-bp fragment with an annealing temperature set at 62 °C. FGD5 protein conservation analysis used NCBI orthologs.

### Model build

The three-dimensional model of the FGD5 protein structure (p.T1078K) was constructed using SWISS-MODEL (https://swissmodel.expasy.org/), and the variant tolerance of protein sites was predicted using the MetaDome website (https://stuart.radboudumc.nl/metadome/) (Wiel et al., 2019).

## Results

### Clinical investigation

**FIGURE 1:**
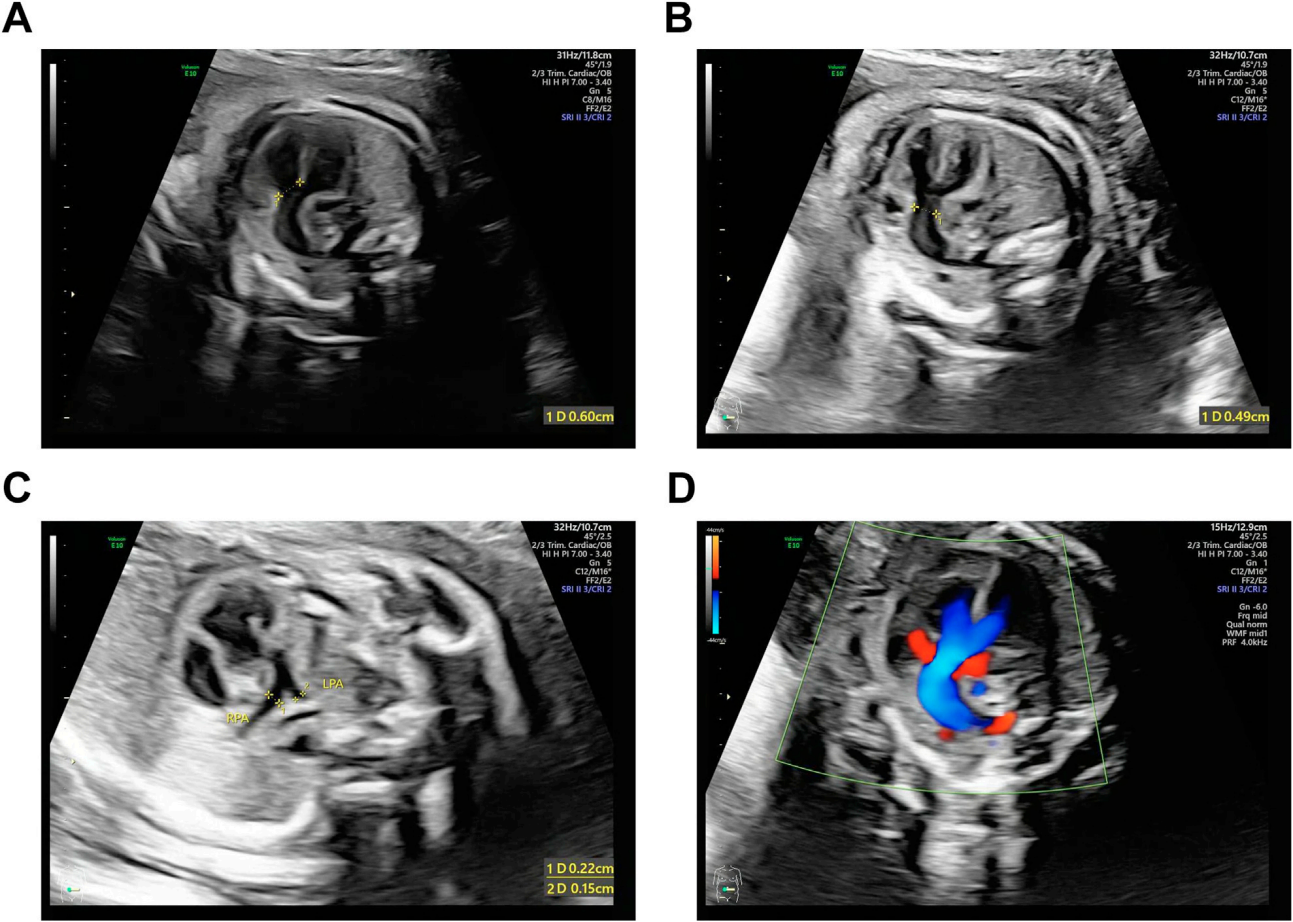
Representative prenatal ultrasound image of a fetus with an *FGD5* variant. **(A)** Interrupted aortic-septal continuity: 0.60-cm defect. **(B)** Aortic luminal diameter: 0.49 cm. **(C)** Pulmonary artery stenosis: left PA 0.15 cm and right PA 0.22 cm **(D)** Systolic right-to-left VSD shunting causing aortic dilation and reduced pulmonary arterial flow.

We enrolled a 26^+3^ weeks’ gestation Chinese fetus (Ⅱ-1) and requested an investigation owing to complex congenital heart malformations (Figure 1). The affected fetus was the only child of non-consanguineous parents. The following physical findings were observed: growth restriction (biparietal diameter, 5.5 cm standard deviation [SD]); head circumference, 21.0 cm (−3.47 SD); and estimated weight, 698 g ± 102 g (−2.84 SD). An ultrasound examination performed at another hospital at 12^+2^ weeks’ gestation demonstrated a crown–rump length (CRL) of 56 mm and nuchal translucency (NT) of 1.2 mm, both within normal limits for gestational age. At 22^+5^ weeks’ gestation, an external ultrasound report noted complex CHD with the features of double outlet right ventricle (DORV), ventricular septal defect (VSD), and suspected pulmonary stenosis, accompanied by microcephaly (head circumference, < −2 SD). At 26^+3^ weeks’ gestation, comprehensive fetal phenotyping was performed at our hospital, including dedicated fetal echocardiography and genetic counseling, to confirm the cardiac diagnosis and evaluate syndromic associations. A grade III four-dimensional prenatal ultrasound identified that the fetus was in a left sacrotransverse position. Fetal head and face evaluation showed a normal oval-shaped calvarial halo, centered midline intracranial structures, no dilatation of the lateral ventricles bilaterally, and appropriately visualized thalami. The cerebellar morphology appeared unremarkable, with visualization of the vermis and absence of significant posterior cranial fossa cistern widening. No disruption in upper lip skin continuity was identified. Fetal spine assessment confirmed intact alignment with regularly spaced parallel echogenic bands. All four extremities showed appropriate development with the visualization of bilateral humeri, ulnae, radii, femora, tibiae, and fibulae; the hands were maintained in physiologic flexion with closed fists, and both feet were fully visualized. Abdominal viscera were appropriately identified, including the liver, gallbladder, stomach, bilateral kidneys without separation, and bladder. Umbilical cord configuration showed two arteries, with no cervical cord indentations noted. The placenta was posteriorly implanted with grade 0 maturity, containing a 3.7 cm × 1.2 cm circumscribed anechoic area exhibiting internal echogenic swirling and no demonstrable flow on color Doppler. Maternal cervical length measured 3.0 cm. Fetal echocardiography highlighted the following findings: (i) four-chamber view with leftward cardiac apex and normal cardiothoracic ratio, concordant atrioventricular connections with a ventricular right-loop configuration, and an intact crux cordis showing normal arteriovenous valve motion without right ventricular hypertrophy; (ii) outflow tract views revealed aortic root dilation (0.49 cm) overriding a 0.6-cm perimembranous ventricular septal defect by 50%, with severe pulmonary stenosis (main PA 0.30 cm, LPA 0.15 cm, and RPA 0.22 cm); (iii) color Doppler showed systolic right-to-left shunting across the ventricular septal defect into the aorta and turbulent accelerated flow through the stenotic pulmonary artery; (iv) venous connections included a single pulmonary vein draining into the left atrium with normal systemic venous return (superior vena cava/inferior vena cava to the right atrium); and (v) a three-vessel-trachea view showed a right-sided aortic arch originating the innominate artery as the first branch, with no detectable ductus arteriosus. These echocardiographic findings led to a diagnosis of fetal heart malformation (TOF) and growth and developmental delay.

### Genetic analysis

**FIGURE 2:**
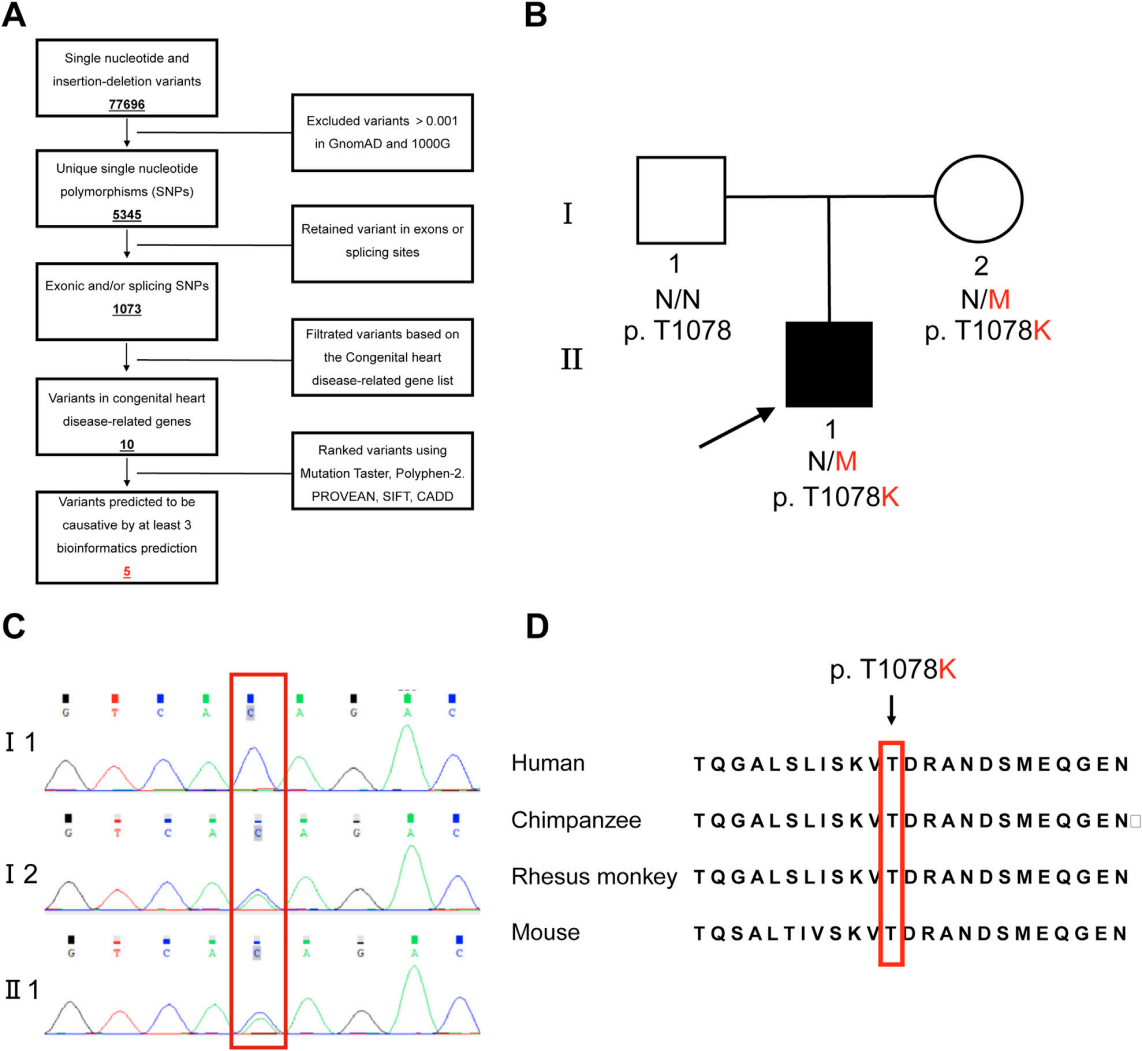
Genetic analysis and variant sit of the *FGD5* gene. **(A)** Strategy of genetic screening in this study. **(B)** Pedigree of a family with a fetus affected by tetralogy of Fallot due to variants on the *FGD5* gene. **(C)** Verification of the *FGD5* variant (c. 3233C>A, p.T1078K) by Sanger sequencing. The red box indicates the variant site. **(D)** Peptide sequences surrounding the mutated residue with multiple interspecies alignments.

Genetic testing was initiated at the parents’ request to identify the underlying causes of the proband’s symptoms. In total, 77,696 variants were detected in the proband, and five variants in five intellectual developmental disorder-related genes were identified according to the strategy (Figure 2A). Four other variants are also present in Table 1. By analyzing the correlations between these five variants and CHD, the phenotypes of *FGD5* and TOF were more consistent. Sanger sequencing verified that the *FGD5* variant (NM_001320276.2: exon9: c. 3233C>A: p.T1078K) in the affected fetus was inherited from his unaffected mother (Figures 2B, C).

**TABLE 1:** Variants identified in the proband by WES in combination with the filtration of intellectual developmental disorder-related genes.

Gene	Variant	Pathogenicity prediction^*^	GnomAD	1000G	OMIM clinical phenotype
*TTN*	NM_133378.4:exon53: c.12659G>C:p.G4220A	MutationTaster: D Polyphen-2: B PROVEAN: D SIFT: B CADD: 1.96 (B) REVEL: 0.555 (D)	0.00077	0.00020	AD, dilated cardiomyopathy-1G; AD, hypertrophic cardiomyopathy-9; AD, myofibrillar myopathy-9 with early respiratory failure.
*RBM20*	NM_001134363.3:exon2: c.325G>A:p.A109T	MutationTaster: D Polyphen-2: D PROVEAN: B SIFT: D CADD: 3.86 (B) REVEL: 0.433 (B)	—	—	AD, dilated cardiomyopathy-1DD.
*FLNC*	NM_001127487.2:exon2: c.469C>T:p.R157C	MutationTaster: D Polyphen-2: D PROVEAN: D SIFT: D CADD: 3.96 (B) REVEL: 0.487 (B)	0.00019	—	AD, cardiomyopathy of the hypertrophic (CMH26); AD, restrictive (RCM5), AD, dilated (CMD1PP), AD, arrhythmogenic right ventricular (ARVD15).
*NTRK3*	NM_001007156.3:exon10: c.995C>T:p.T332M	MutationTaster: D Polyphen-2: D PROVEAN: D SIFT: D CADD: 2.94 (B) REVEL: 0.451 (B)	0.00014	0.00020	Congenital heart defects, ventricular septal defect.
*FGD5*	NM_001320276.2:exon9: c.3233C>A:p.T1078K	MutationTaster: D Polyphen-2: D PROVEAN: D SIFT: D CADD: 3.70 (B) REVEL: 0.292 (B)	—	—	AD, Tetralogy of Fallot

D, disease-causing; B, benign; AD, autosomal dominant.

*, Pathogenicity prediction of CADD and REVEL are based on the paper PMID: 36413997.

The *FGD5* variant (c. 3233C>A: p.T1078K) was *de novo* (PS2) and absent in gnomAD and 1000 Genomes (PM2). It was predicted to be disease-causing using the following tools: MutationTaster, PolyPhen-2, PROVEAN, SIFT, CADD, and REVEL. Based on the assessment using these six pathogenicity prediction tools for missense variants, four tools categorized this variant as pathogenic, and the amino acid site p.T1078 was highly conserved evolutionarily (Figure 2D, PP3). Thus, based on the ACMG standards and guidelines (PS2, PM2, and PP3), we determined the variant to be “likely pathogenic” and responsible for the symptoms of the proband.

**FIGURE 3:**
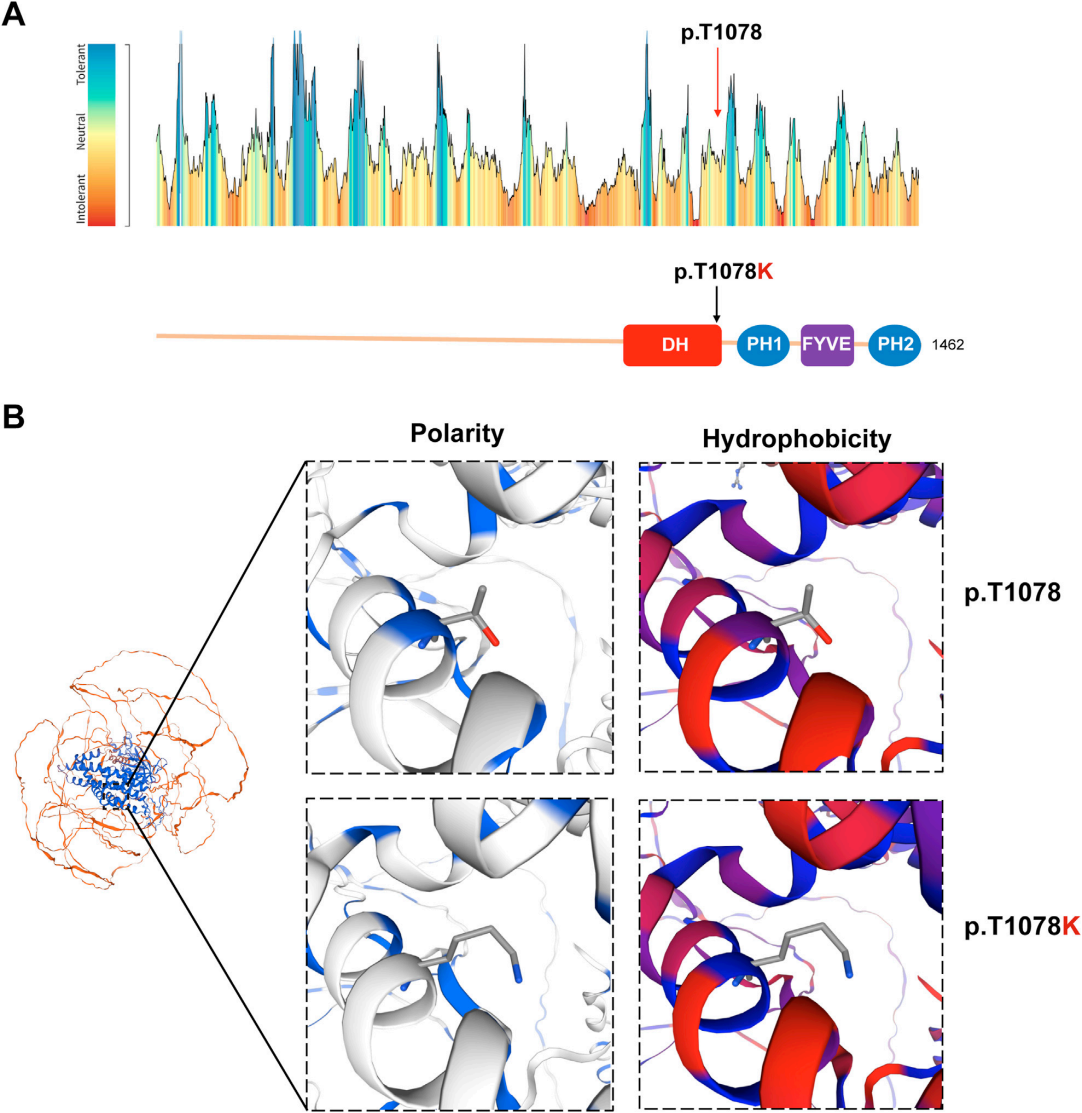
Normal and variant structures of the FGD5 protein. **(A)** Intolerance of FGD5 to functional genetic variation predicted by MetaDome and the position of variants within the DH motif. The red arrow indicates the variant site in this study. **(B)** Three-dimensional model analysis of polarity and hydrophobicity of wild and mutant types of the FGD5 protein.

Based on MetaDome analysis, the variant site was predicted to be neutral, favoring the occurrence of functional genetic variations (Figure 3A). Furthermore, the three-dimensional protein model reveals that the p.T1078K variant is located within an α-helix. This substitution replaces threonine (Thr, T) with lysine (Lys, K), significantly altering the amino acid’s polarity and hydrophobicity—specifically, a shift from threonine to lysine typically involves decreased hydrophobicity and increased hydrophilicity. These physicochemical changes are predicted to substantially enhance the potential pathogenicity of this point variant (Figure 3B).

## Discussion

To date, six members of the FGD protein family (FGD1–FGD6) have been identified, all of which exhibit high structural conservation. These proteins specifically recognize phospholipids such as phosphatidylinositol bisphosphate (PIP2) and phosphatidylinositol 3-phosphate (PI3P), along with GTPases, functioning as Rho GEFs to regulate cellular development (Snyder et al., 2002). Structurally, FGD proteins contain an N-terminal low-conservation disordered region, followed sequentially by a Dbl homology (DH) domain, two pleckstrin homology (PH) domains (PH1 and PH2), and a C-terminal FYVE domain. The DH and PH1 domains together form a canonical Rho GEF module—the DH domain catalyzes GTP/GDP exchange activity, while PH1 mediates protein localization and activation. Both PH1 and PH2 domains modulate interactions with phosphoinositides (PIs), and the FYVE domain serves as a specific receptor for PI3P. The DH domain adopts a unique “armchair-like” structure composed of six α-helices, with conserved residues in CR1, CR3, and the C-terminal α-helix 6 forming the critical interface for GTPase binding. Amino acid substitutions in these regions significantly impair GEF activity (Liu et al., 1998). In this study, we identified a missense variant (p.T1078K) located within α-helix 6 of the DH domain, which is predicted to severely disrupt FGD5–GTPase interactions. Clinical studies have established strong associations between the FGD family protein variants and human diseases. For example, missense variants in the DH domain of *FGD1* cause Aarskog–Scott syndrome (Orrico et al., 2004; Pérez-Coria et al., 2015), while the DH domain variants in *FGD4* lead to Charcot–Marie–Tooth disease type 4H (CMT4H) (Hyun et al., 2015). These findings underscore the essential role of DH domain conservation in maintaining proper protein function and preventing genetic disorders.

As hereditary TOF generally manifests as an autosomal irregular dominant disorder, studies consistently report asymptomatic carrier status among substantial numbers of probands’ parents and first-degree relatives (Goldmuntz et al., 2001; Bauer et al., 2010; Baban et al., 2014). In this study, the affected fetus exhibited typical imaging phenotypes of TOF on cardiac ultrasonography, while the mother carrying the variant showed no TOF phenotype. This observation aligns with the characteristic irregular dominance of inherited TOF. Any disruption in the angiogenesis process can lead to vascular abnormalities, resulting in the development of congenital heart disease (Jin et al., 2024). Based on current research findings, the FGD5 protein may regulate vascular pruning by participating in the VEGF (vascular endothelial growth factor) signaling pathway and activating the CDC42 protein, thereby modulating neovascular networks in both physiological conditions and tumor tissues (Farhan et al., 2017; Heldin et al., 2017; Valla et al., 2018). Moreover, evidence indicates that complete deletion of the *FGD5* gene leads to embryonic lethality, accompanied by impaired cardiac and/or vascular development (Cheng et al., 2012; Gazit et al., 2014). Owing to the high penetrance of *FGD5* variants (Reuter et al., 2020), genetic counseling and prenatal diagnosis should be recommended for the proband’s parents, considering subsequent pregnancies.

## Conclusion

We detailed the echocardiographic findings of a male fetus at 26^+3^ weeks of gestation. We identified a novel *FGD5* variant (c. 3233C>A, p.T1078K) in him and analyzed the pathogenicity using three-dimensional protein modeling. Our report provides a perspective on the progress of *FGD5*-related diseases and extends our understandings of *FGD5*-related symptoms. Our identification and compilation expand the pathogenic variant spectrum of *FGD5*, suggest a linkage between *CDC42* and the *FGD5* variant in the DH domain, and contribute to molecular diagnosis and genetic counseling in patients with *FGD5* variants.

## Data Availability

The WES datasets presented in this study can be found in online repositories China National Center for Bioinformation (https://www.cncb.ac.cn/), accession number PRJCA047101.
